# Bioengineering of Functional Nanosilver Nanogels for Smart Healthcare Systems

**DOI:** 10.1002/gch2.201800044

**Published:** 2018-08-17

**Authors:** Sadiya Anjum, Bhuvanesh Gupta

**Affiliations:** ^1^ Bioengineering Laboratory Department of Textile Technology Indian Institute of Technology New Delhi 110016 India

**Keywords:** bacteria, biomaterials, healthcare, infections, nanogels, nanosilver

## Abstract

Functional designing of nanogels has become an attractive domain of biomedical engineering to develop bioactive materials with innovative features for the human healthcare system. Nanosilver has attracted enormous attention due to its wide antimicrobial spectrum and ability to kill almost all types of bacteria in its vicinity. However, the most crucial challenge for bioscientists is the lack of binding ability of nanosilver with the material surfaces that allow nanosilver to leach out to the surrounding tissue and exert toxicity while the biomaterial is in contact with the living system. Designing nanosilver within a nanogel confinement offers enormous possibilities to develop functional bioactive nanoparticles that may be bonded to any biomaterial surface via the nanogel functionality. This approach requires the proper combination of material science with nanotechnology and biotechnology to innovate interesting domain of functional nanogels with unique features. This work aims at providing a critical review on the current progress, approaches, and vision in designing nanosilver‐entrapped nanogel particles with diverse functionality, and their bioactivity against microorganisms for human healthcare devices.

## 1 Introduction

**Figure 1:**
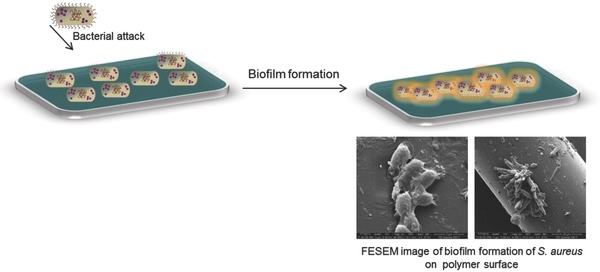
Schematic presentation of bacterial growth on a material surface.

Infection has become a serious problem the world over and has been the focus of sincere efforts toward developing a functional system against microbial infection. The problem sometimes becomes critical and may eventually lead to the formation of biofilm due to the adherence of bacteria where secretion as well as accumulation of extracellular products takes place (**Figure** **1**). As a consequence of this, the infection spreads to the surrounding tissues and becomes responsible for the high rate of clinical complications. Situation becomes more complicated with the emergence of drug‐resistant pathogens, such as methicillin‐resistant *Staphylococcus aureus* (MRSA), macrolide‐resistant *Streptococcus pyogenes*, penicillin‐resistant *Streptococcus pneumoniae*, vancomycin‐resistant *Enterococcus, Escherichia coli (E. coli), Klebsiella pneumoniae, Shigella flexneri, Salmonella enterica, Acinetobacter baumannii, Pseudomonas aeruginosa (P. aeruginosa), Vibrio cholerae*, and multidrug‐resistant Mycobacterium.^1^, ^2^, ^3^ As a consequence, the bacterial attachment adversely affects the organ functionality and decreases the lifetime of the patient and sometimes becomes fatal as well. Enormous research efforts are being made to develop materials that effectively deal with such a bacterial invasion and act against them thereby preventing biofilm formation on a biomaterial surface.^4^, ^5^, ^6^, ^7^

Nanotechnology has emerged as a fascinating domain of biomedical engineering where advanced materials and biomedical devices with innovative features may be fabricated.^8^, ^9^, ^10^ The application of nanomaterials ranges from the novel domain of drug targeting to the creation of the infection‐resistant surfaces.^11^ With the help of nanocarriers, the bioactive agent may be selectively transmitted to the intracellular pathogens in a very controlled manner.^9^ In the recent days, nanosilver particles have been recognized as optimal candidates for defeating pathologies, previously treated with conventional antibiotics, because of their strong and broad‐spectrum antimicrobial characteristics.

**Figure 2:**
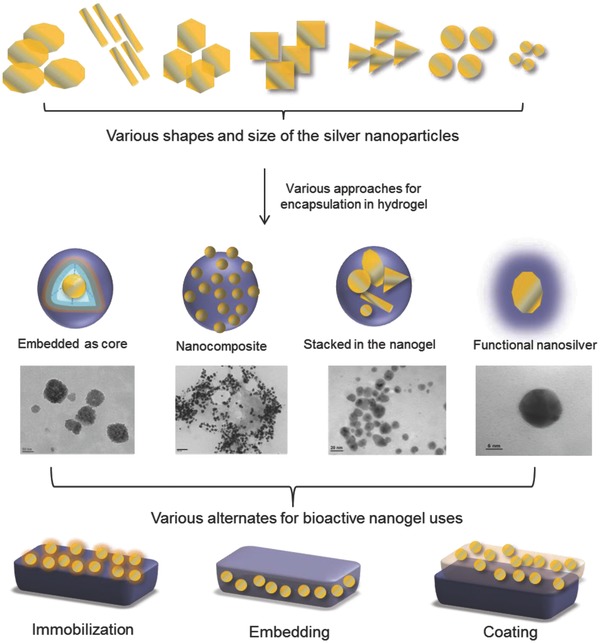
Architecture of nanosilver nanogel and various alternatives for their uses.

Silver has been very effective against a broad range of aerobic bacteria, anaerobic bacteria, yeast, filamentous fungi, and viruses, and has made significant contribution to developing antimicrobial systems for wound and burn care products.^12^ However, silver ions are easily solvated in the aqueous system and hence get detached from the matrix thereby leading to the sharp reduction in the antimicrobial efficiency of the material. Several approaches are available for the preparation of nanosilver particles.^13^, ^14^, ^15^, ^16^ Nanoparticles of different shapes and sizes may be immobilized, coated, and embedded onto a polymer surface so that antimicrobial materials with infection‐resistant features may be designed (**Figure** **2**). Efforts have been made to incorporate silver into nano or microparticles of the hydrogels for various applications.^17^ However, such a microparticle may lead to fast release of silver from the matrix leading to very short‐term effectiveness, comparatively, nanoparticles. Different approaches for microbial infection management using nanomedicines have been nicely reviewed recently.^11^ A recent report, microphages and lung epithelial cells were exposed to silver nanoparticles with different stabilizing agent, indicating membrane damage and loss of integrity. These results suggested that the particle morphology has significance in dictating the cytotoxicity of the nanoparticles. The authors claim that the positively charged particles have great affinity toward cell membrane penetration and cellular interualization.^18^ It may therefore be visualized that the microbial infection management requires a very smart planning and execution using the knowledge of material science, bioscience, and nanoscience together.

## 2 Functional Bioengineering of Nanogels: State of the Art

**Figure 3:**
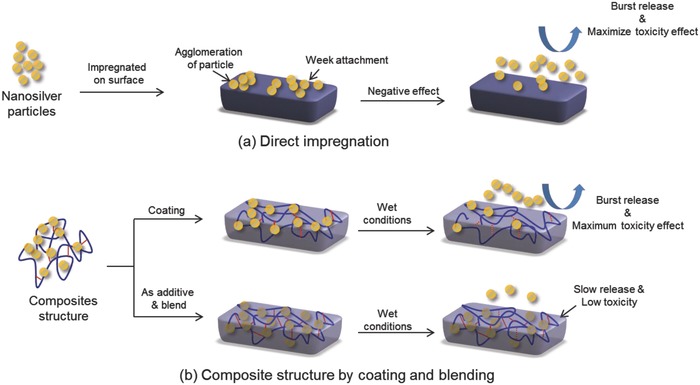
Schematic representation for the application of nanosilver particles: a) direct impregnation and b) coating and blending approaches.

Nanosilver particles may be directly impregnated on a material surface where no interacting site is available for adherence of the particles. Unfortunately, this leads to the weak attachment of silver with the material surface leading to the fast leaching out and its subsequent toxicity to the surroundings (**Figure** **3**a). In an alternate option, nanoparticles may be trapped within the hydrogel matrix by developing a nanocomposite structure.^19^, ^20^ In both cases, the adverse effect of nanosilver may be observed in terms of low binding, burst release, and cytotoxicity (Figure 3b). Therefore, few crucial factors need to be taken into consideration toward the use of nanosilver for the development of antimicrobial materials. The first one is the inert nature of the nanosilver. Second, the lack of functionality as the anchoring site to avoid free diffusion across biological barriers and penetration into cells and tissues.^13^, ^14^, ^16^ Therefore, the real challenge lies in designing and creating functional nanosilver that would bind to the biomaterial surface.

**Figure 4:**
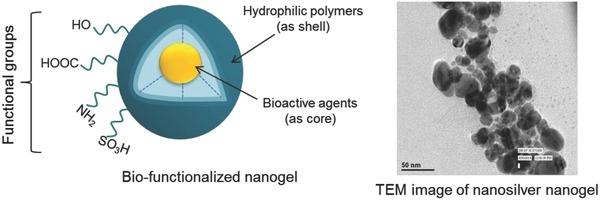
Representation and TEM image of a functional nanogel.

Functional nanosilver has become the subject of enormous interest in the world of bioactive nanogels.^21^, ^22^, ^23^, ^24^ Polysaccharides may be capped with nanosilver to achieve antibacterial nature where cytotoxicity toward the mammalian cells is significantly reduced. Confocal imaging of actin and tubulin cytoskeleton suggested that capped particles do not affect the mammalian cells even at higher concentration.^25^ This suggests that the nanogel requires precise control of its physicochemical structure in order to achieve desired biological features in the material. The beauty of the nanogel is that one can design the biomaterial in terms of functionality and structure, which may suit specific application (**Figure** **4**). Whatever the alternative we opt for, a biocompatible layer is created as the sheath around the silver nanoparticle. Hydrogel sheath would not only encapsulate the silver nanoparticle but also create binding sites to anchor biomaterial surfaces. This is a smart move where biocompatible polymer sheath stays in contact with tissues and minimizes the adverse cytotoxic effect of direct contact of the silver nanoparticle.^26^, ^27^ The functionality present in the hydrogel seems to provide advanced features to the nanogel particles. With the help of nanocarriers, the nanosilver particles may be selectively transmitted to the intracellular pathogens in a very controlled manner. A combination of the biofunctionality and nontoxicity makes nanogel a fascinating material and hence opens up enormous possibilities of designing and engineering them for biomedical applications.

**Figure 5:**
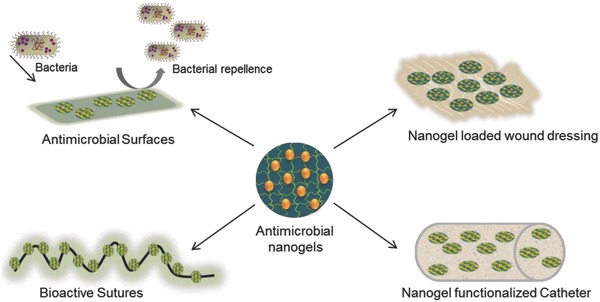
Application of functionalized nanogels for infection control.

The bioengineering requires incorporation of specific functional groups that would allow nanogel to anchor and to bind to a substrate to achieve bioactivity. The state of the art in nanogel engineering conceptualizes that the functional groups would act as the anchoring sites either for the immobilization of biomolecules or for the interaction with a material surface by ionic, covalent, coordinate or by hydrogen bonding while protecting the biocompatibility with the surrounding tissue. These unique features of a nanogel therefore open up enormous possibilities in developing sutures, implants, wound dressings, and infection‐resistant surfaces (**Figure** **5**).^28^, ^29^, ^30^, ^31^, ^32^, ^33^, ^34^

## 3 Designing of Functional Nanosilver within Nanogel

**Figure 6:**
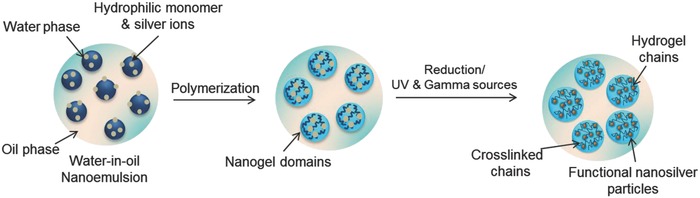
Preparation of functional nanosilver by the nanoemulsion process.

The realization of bioactive nanogels has become the subject at the forefront of bionanotechnology. The crucial aspect of the nanogel preparation is the size and chemical functionality, where nontoxicity and bioactivity is still protected. Nanoemulsion process has shown enormous potential toward the nanoparticle preparation. However, there are more possibilities of designing the chemistry of nanostructures during the polymerization process. The most appropriate route is the polymerization of a monomer as water phase in water‐in‐oil (w/o) nanoemulsion in the presence of silver nitrate, followed by the reduction of silver ions into nanosilver particle (**Figure** **6**). Alternatively, a polymeric hydrogel may be used in the water phase in w/o nanoemulsion where nanosilver particle is generated in situ. Both of these approaches have very favorable features where a nanosilver nanohydrogel particle is formed with a functional nanogel surface. Various approaches have been used for the preparation of nanogels, i.e., radiation process, electrochemical process, thermal reduction process, and chemical reduction. However, the recent trend has been dedicated more toward the radiation and chemical processes. A wide range of functional groups, such as carboxyl, amide, hydroxyl, amino, and sulfonic, may be created on the nanogel surface by using appropriate monomers.

### 3.1 Radiation Process

**Figure 7:**
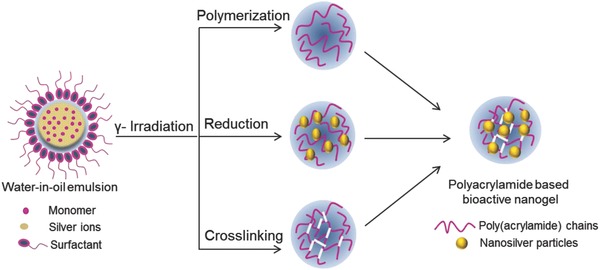
Schematic representation of the development of bioactive nanogels by gamma reduction. Reproduced with permission.^28^ Copyright 2017, VBRI Press.

Radiation process seems to be the smart approach as compared to the other approaches. The radiation performs three simultaneous processes, i.e., polymerization, crosslinking, and silver reduction, but independent of each other. The radiation initiates polymerization of the monomer in a w/o nanoemulsion where a macromolecular chain is formed. Second, it tends to crosslink the polymer chain as soon as it is formed in the system. Third is the reduction of the silver ions so that the nanosilver formation within the nanogel matrix takes place. In addition to it, the radiation polymerization leads to the extremely pure nanogel without any contamination that may arise due to initiators in a chemical process. These three processes therefore influence the process of nanogel formation (**Figure** **7**).^28^ This nanohydrogel matrix contains functional groups so that the resultant nanogel may bind to a biomaterial surface via hydrogen bonding, ionic interaction, or by covalent linkages. The indirect binding of the nanosilver to a biomaterial surface via nanogel linkage therefore reduces the toxicity level of free silver ions.

**Figure 8:**
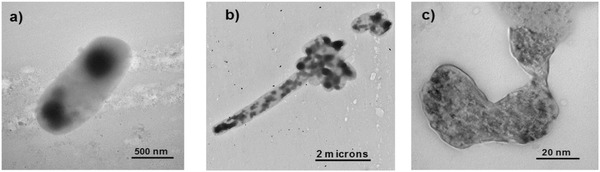
TEM images of *E. coli*. a) Control bacteria sample. b,c) Bacteria samples that were previously treated with silver nanoparticles. Reproduced with permission.^28^ Copyright 2017, VBRI Press.

**Figure 9:**
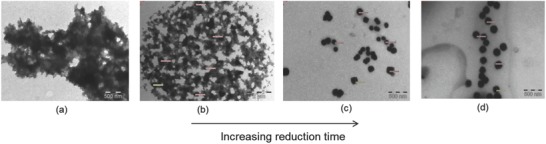
TEM images of PMAA nanosilver nanohydrogel with the variation of reduction time: a) 30 min, b) 2 h, c) 4 h, and d) 6 h. Reproduced with permission.^32^ Copyright 2016, Elsevier.

The synthesis of polymethacrylic acid nanogels with nanosilver core has been carried out by the radiation process where well‐dispersed and stable nanoparticles in the range of 10–25 nm were formed.^34^ Similar study with polyacrylamide nanogel showed excellent hydrogel character along with the broad antimicrobial efficiency against both *E. coli* and *Staphylococcus aureus* (*S. aureus*).^28^ It was observed that the cell wall of bacteria ruptures in contact with the nanogel and the leakage of saccharides and proteins across bacterial cell wall follows (**Figure** **8**). These bioactive nanogels may be used in combination with essential oils for the development of infection‐resistant surfaces for face wipes, baby diapers, and other skin‐contacting materials.^31^ Synergistic effect of bioactive nanogels and essential oils has been monitored by histological evaluation. It seems that such a material is suitable as infection‐resistant material for skin care. Alternatively, it is possible to use a two‐step process where the radiation polymerization in a w/o nanoemulsion is carried out to prepare nanogel particles. In the second step, the reduction of silver ions is accomplished by using either radiation or any suitable reducing agent. The two‐step process provides a better control over the nanogel molecular weight and the degree of crosslinking.^32^ Gamma radiation has been used for the crosslinking of the nanogel and in situ reduction of the silver ions to nanosilver (**Figure** **9**). As the reduction time increases, the dimensional stability due to the crosslinking of the network is achieved. After 6 h of reduction time, highly stable and smaller size nanoparticles are formed (Figure 9d).

**Figure 10:**
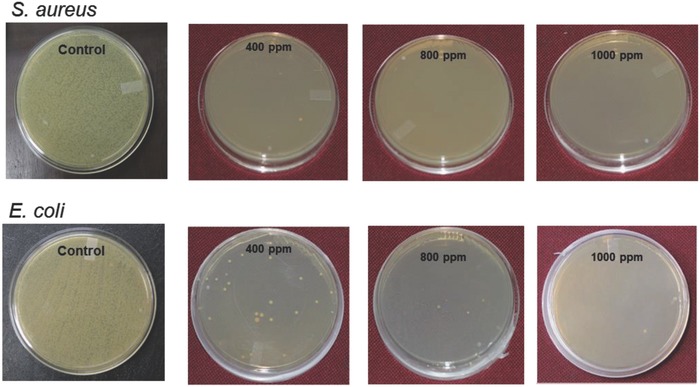
Antimicrobial nature of nSnH at different concentrations. Reproduced with permission.^32^ Copyright 2016, Elsevier.

The base matrix, in fact, acts as the carrier for the nanosilver particles along with the possibility of biointeraction with a surface via its functional groups. The antimicrobial efficiency of these nanogels was monitored with varying concentration of nanogels and it was found that a very small amount of bioactive nanogel is sufficient for the bacterial reduction (**Figure** **10**). These nanogels retain their antimicrobial nature in spite of the fact that the nanosilver remains encapsulated within the hydrogel matrix. The antimicrobial nature enhances as the nanogel content increases from 200 to 1000 ppm. A minimum concentration of 600 ppm has been projected to achieve complete reduction against *S. aureus* and *E. coli*.

**Figure 11:**
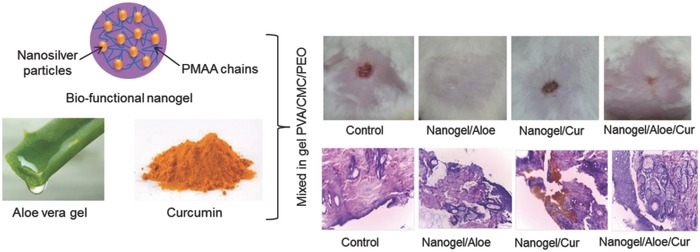
Wound‐healing studies of biofunctionalized nanogels with *Aloe vera* and curcumin‐based wound dressings. Reproduced with permission.^32^ Copyright 2016, Elsevier.

These nanogels may be combined with other biological moieties such as *Aloe vera* and curcumin as coating material to develop herbal composite dressings where excellent wound healing features were observed. It is interesting to see that nanogel in combination with *Aloe vera* performs much better than its combination with curcumin (**Figure** **11**). May be, the *Aloe vera* has better chemical composition and ingredients that favor the healing process. Alternatively, chitosan (CS) and silver nanogel combination may also be synthesized by gamma radiation where CS helps in the stabilization of these particles.^35^ The nanoparticles obtained via this process remain stable for more than three months any phase separation. Moreover, they could be easily anchored as antimicrobial material due to the presence of CS layer around the particle.

The reduction and fragmentation of silver nanoparticles by applying ultraviolet (UV) radiation is another chemical‐free approach, where silver ions are reduced due to the presence of hydrated electrons, produced via UV irradiation. The UV‐radiation source transforms the silver ions into smaller sizes of nanoparticles with uniform distribution with good antimicrobial activity against *E. coli*, *S. aureus*, and MRSA.^36^ Free radical polymerization is another interesting alternative in terms of the wide choice of the initiators and the nanodimensions of the particles.^28^, ^34^ The monomer along with the bioactive moiety may be taken in the water phase in a w/o nanoemulsion. The stability of the nanoemulsion would depend on the composition of the system involving water phase, surfactant type, and the nature of the bioactive additive. By precise consideration of the chemical structure of monomers and their eventual combinations within a nanoemulsion, novel architecture of the nanogel may be achieved. The physicochemical properties of the nanogel may be controlled by monitoring crosslinking density and solvent effect so that polymerization takes place leading to the nanogel entrapping the bioactive molecule. However, interpenetrating network structures may also be obtained while a monomer is polymerized within the polymeric water phase. In w/o nanoemulsion, acrylic acid (AA) forms 3D structure within gelatin domain in the presence of *N,N′‐*methylene‐*bis*‐acrylamide crosslinker, leading to a network structure of the nanogel.^37^ The water absorption by the nanogel is certainly very important, which would help in the release of bioactive molecules. The modified microemulsion process was used for the preparation of polyvinyl pyrrolidone (PVP) nanogels where silver and other metal ions are induced in the nanogel by the diffusion process, followed by the reduction in sodium borohydride solution. Furthermore, positive charges may be created on PVP nanogels by quaternization with hydrochloric acid and 2‐bromoethylamine on pyridine moieties in the nanonetwork. These functionalized nanogels and their composites have been investigated as a potential antimicrobial agent against different bacterial strains.^38^

**Figure 12:**
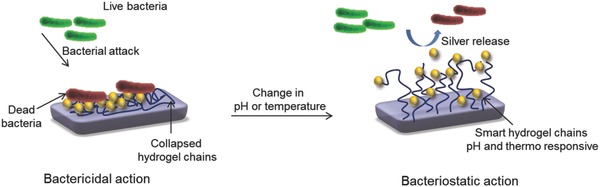
Bactericidal and bacteriostatic action of the stimuli‐responsive smart functional nanogels.

An additional feature of the micellar polymerization has been the preparation of smart nanogels. By virtue of the presence of the AA and MAA, the nanogels offer pH sensitivity leading to the quick transition at pH > 4.6.^39^ The nanogel based on poly(methyl methacrylate‐*co*‐acryloyl phenylalanine) offers pH‐responsive nature while encapsulating the nanosilver within its domain for antimicrobial action against *Bacillus* and *E. coli*.^40^ The design and preparation of silver‐loaded copolymer vesicle of polycaprolactone‐polyacrylic acid was reported.^41^ In copolymer vesicle, the pendent group has variable pKa values due to interaction of each block and helps in the growth of nanosilver particles inside the core of vesicle.^41^ These stimuli responsive nanogels show sharp noticeable transition in properties and action at altered stimuli conditions. At stimulate conditions, the change in release behavior of trapped bioactive nanoparticles is observed, which influences the bacteriostatic and bactericidal properties (**Figure** **12**). These nanogels are used for coating as well as additives for the development of smart material for infection‐resistant surfaces and show excellent antimicrobial efficacy with lower concentration.

### 3.2 Chemical Process

**Figure 13:**
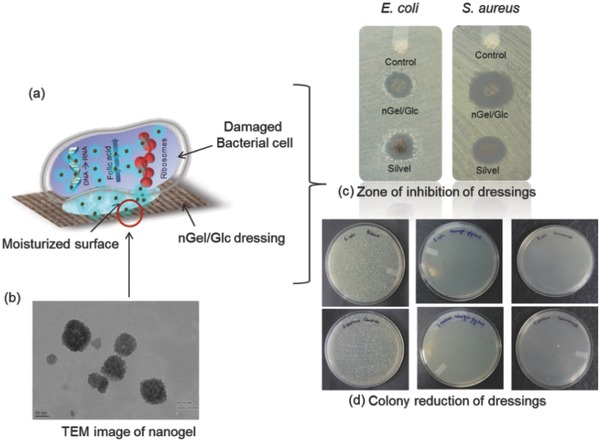
a) Schematic representation of antimicrobial action of nGel/Glc dressing and Silvel (commercial dressing); b) TEM image of nanogel; c) antimicrobial nature of dressing by zone of inhibition; and d) colony reduction against *E. coli* and *S. aureus*.

Advancement in the nanogel preparation is the use of polymers instead of using monomers and subsequently polymerizing them. These polymers may range from synthetic ones, such as polyvinyl alcohol (PVA), PVP, and polyethylene glycol (PEG), to biodegradable, such as CS, pectin, dextran, gelatin, alginate, and cellulose.^42^, ^43^, ^44^, ^45^, ^46^, ^47^ This is the smarter way of preparing nanogels by preselection of an appropriate polymer and hence a proper control over the nanogel features may be achieved. Eventually, a mixture of the polymers may also be used to have a more focused application area for the nanogel. Chemical reduction of silver is carried out in two different manners, such as in situ and ex situ growth of nanoparticles within the gels. In the in situ approach, both reducing and stabilizing agents work simultaneously, resulting in a core–shell‐type structure of the bioactive nanogels. While in the ex situ approach, silver nanoparticles are separately prepared and introduced in between the gels for the formation of nanocomposite structure. PVA exhibits good biocompatibility and nontoxicity along with excellent hydrophilic in nature. PVA is also used as stabilizing gel matrix for the in situ growth of silver nanoparticle within the nanogel using fructose as a reducing agent. The combination of the bioactive nanogel with glycerol offers advantage in terms of the antimicrobial as well as moist healing as observed in animal models (**Figure** **13**). Bioactive PVA nanogel in combination with glycerol (nGel/Glc) dressing has been very effective in inhibiting scar formation significantly and leads to faster healing of the wound.^45^ Alternatively, nanosilver may be capped by thioethylene amine so that the nanosurface is functionalized into aminated structure. This was subsequently immobilized on to polylactic acid (PLA) surface by carbodiimide linkage.^48^

PEG is an interesting polymer due to its biocompatibility and biofouling features that help in infection control in contaminated wounds. PEG‐nanosilver particles may be prepared by dual action of PEG matrix, i.e., PEG helps in silver reduction into nanosilver while at the same time, it provides hydrogel sheath and stabilizes the nanosilver particles. The resultant nanogel therefore contains a layer of PEG around nanosilver that may offer an anchoring moiety with a biomaterial surface. It is observed that the PEG bioactive nanogels may be immobilized by hydrogen bonding onto plasma‐treated polypropylene or polyester filament surface to develop an antimicrobial suture.^29^, ^49^ Plasma treatment using carbon dioxide (CO_2_) as the carrier gas generates carboxyl groups while oxygen plasma creates hydroxyl groups along the macromolecular chains.^50^ In both the cases, PEG bioactive nanogel may anchor via hydroxyl group to the biomaterial surface by hydrogen bonding with the hydroxyl and carboxyl functional groups.

The in‐situ preparation of nanosilver within epichlorohydrin crosslinked carboxymethyl chitosan (CMCS) nanogels was reported.^51^ In such nanocomposites as well, gel plays dual functionality, where it acts as the reducing agent and also stabilizes silver nanoparticles. Subsequently, these nanogels show excellent antimicrobial activity against different types of bacterial strains. Similarly, Ferrer et al. reported the development of polysaccharide‐based nanogels with embedded silver particles and exhibit excellent antimicrobial activity.^52^ Nanogel comprises dextran and lysozymes, where lysozymes help in nucleation and stabilization of silver nanoparticles with in the nanostructured gel. Cryo‐Transmission electron microscopy (TEM) investigation of the nanoparticles showed that the majorly of the particles were in the range of 2–5 nm and spherical in shape. These nanogels show size‐dependent antimicrobial activity, i.e., smaller particles exhibit larger surface area and hence increased interaction as compared to the larger nanoparticles.

Recently, electrospun polyurethane/keratin and nanosilver have been developed for the wound healing applications, where silver nanoparticles were ex situ grown over the electrospun mat after dipping into ascorbic acid solution.^53^ Scanning electron microscopy (SEM) images show the growth of nanoparticles onto the electrospun web with different concentration of ascorbic acid. This nanomat shows excellent antimicrobial results without reducing the cytocompatibility of the matrix. Alternatively, CS, PEG, and sodium borohydride may be mixed together for the reduction and stabilization of nanosilver particles within the CS‐PEG gels. The presence of oxygen and nitrogen groups in PEG and CS, respectively, ensures the stabilization of nanoparticles via direct bonding with these electron donor sites and provides steric protection due to bulky polymeric network. The antibacterial activity of nanocomposite against *E. coli* shows good zone of inhibition.^54^ CS with different molecular weights and their sodium salts was employed for the reduction of silver ions within the gel. Broad distribution of nanoparticles was observed in the size range of 20–175 nm of silver–chitosan nanocomposites. The antibacterial activity of nanocomposite was evaluated against *S. aureus* using the disc diffusion method.^55^ Silver nanoparticles within CS‐glycerol gel have been prepared by using gallic acid as reducing agent, and very small size of nanoparticles is observed and shows good inhibition zone against *Streptococcus mutans*.^56^ Biopolymers, such as carboxymethyl cellulose (CMC), pectin may also be used to develop functional nanosilver with in the nanosize gel matrix. Both CMC and pectin may be oxidized by periodic acid so that dialdehyde functionality is created in the existing gel. These aldehyde groups would in situ reduce the silver ions into nanosilver. Subsequently, these nanocomposites were blended with gelatin where the remaining aldehyde group in the matrix led to the crosslinking with polymer resulting into a material for potential use for wound dressing.^57^

Smart and responsive nanogels add to the attraction of nanotechnology based healthcare systems, as they exhibit enormous potential to adapt the surrounding environment. Core shell silver nanoparticles have been prepared by using oleic acid and ferric ions using emulsion polymerization.^58^ This led to the formation of mono disperse nanogels by precise control of crosslinking of the particles. Nanogel preparation was further extended to other monomers, such as styrene, acrylamide, methyl propane sulfonic acid, and N‐Isopropylacrylamide (NIPAm) to get core–shell structure. This opens up a beautiful domain where hydrophilicity of silver nanoparticles may be controlled by proper choice of the monomer. While oleic acid gave contact angel of ≈85°, the shell composition of styrene/2‐acrylamido‐2‐methyl‐1‐propane sulfonic acid (AMPS)/NIPAm offered a very low contact angle of ≈26° showing a highly hydrophilic morphology. NIPAm‐based nanosilver nanogels have been investigated in combination with methacrylic acid using ammonium persulfate as the initiator.^30^ Spherical morphology of nanogels was monitored by SEM analysis where the dimensions varied between 180 and 200 nm (slightly higher than the nanocount of <100 nm). These nanogels were coated on to the fabric and were observed to be effective against *Staphylococcus epidermidis* and *E. coli* suggesting a prominent material within the textile industry as antimicrobial surgeon's care and patient drapings. Similarly this approach has been followed for hybrid core–shell structure where nanosilver remains surrounded by NIPAm‐AA copolymer shell.^39^ However, the size of nanoparticles has been controlled to 40–80 nm. This nanogel has been projected toward tumor cell imaging and local delivery due to the fact that the nanogel is capable of crosslinking the cellular barrier thereby offering both optical and therapeutic functionality.

### 3.3 Miscellaneous Approaches

Some approaches have found way into functional nanosilver preparation in spite of the fact they are very rare in use. CMCS with polyethylene oxide (PEO) was used for the nanosilver composite, in which CMCS played dual functionality of reduction as well as stabilization of nanoparticles. The CMCS‐Ag‐PEO nanocomposite is highly effective antimicrobial material with zone inhibition against *S. aureus*, *P. aeruginosa, E. coli*, and *Candida albicans*.^59^ A nanocomposite of CS and PVP with silver was prepared by the thermal reduction process where silver ions were coordinately bound to hydroxyl and amino groups of CS.^60^ Microwave irradiated in situ synthesis of nanosilver particles within Polyacrylic acid (PAA)/PEG hydrogel was observed where sodium citrate salt was used for the reduction of silver ions.^16^ Silver nanoparticles were entrapped within the hydrogel structure due to the presence of carboxylate anions in PAA chains and additionally stabilized by PEG. Nanosilver‐decorated lipase‐sensitive polyurethane micelles have been reported.^61^ This nanocomposite structure consists of PCL as hydrophobic core and hydrophilic PEG shell where nanosilver particles are placed. Interestingly, the PCL core of polyurethane (PU) micelles degraded and released the decorated silver nanoparticles in the presence of lipase, which resulted in excellent antibacterial activity of the nanocomposites with minimum toxicity against mammalian cells. In similar study, the growth of octahedral‐shaped nanosilver particle within PCL matrix was reported for antibacterial bone scaffolds.^62^ The nanocomposite as scaffold exhibited localized antibacterial activity without any adverse effect on viability of human fetal mesenchymal stem cells.

## 4 Future Perspectives

**Figure 14:**
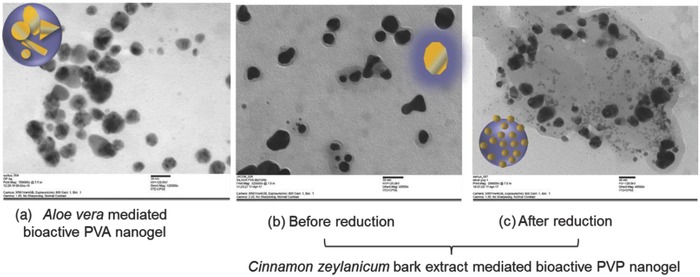
TEM images of herbal extract mediated bioreduction of nanosilver. a) *Aloe vera* gel. b,c) *Cinnamon zeylanicum* bark extract.

The reduction of silver ions into nanosilver has led to the development of several commercial products.^62^, ^63^, ^64^, ^65^ The reduction step in nanotechnology is very important. Inorganic salts are used for the reduction process but sometimes the unreacted salts cause toxicity to the final product. However, the organic molecules such as fructose and ascorbic acid show prominence not only due to their natural origin, but also behave as excellent reducing agents. The herbal extracts mediated synthesis of nanoparticles seems to be a better and ecofriendly approach. The herbal extracts have wide perspective for the bioreduction of silver particles and stabilized with biocompatible hydrogel, which may be termed as functional bioactive nanogels. In recent studies, *Aloe vera* gel extract has been used for the bioreduction of the silver ions in the presence of PVA as capping and stabilizing agent (**Figure** **14**a). The presence of nanosilver in the core part was confirmed by the energy dispersive X‐ray analysis that is coupled with TEM instrument. In another study, *Cinnamon zeylanicum bark* extract was employed for the green mediated synthesis of silver nanoparticles in the presence of PVP gel matrix. In this study, the pre and postaddition of PVP hydrogel in the reaction medium was monitored where the influence of the addition of PVP in the appearance of nanoparticles was observed. When the polymer matrix is added before the reduction process, the core–shell morphology of structure was observed. When the polymer matrix was added after the reduction process, composite structure was observed in TEM images suggesting the presence of nanoparticles within the hydrogel matrix (Figure 14b). Such an approach shows advancement in the nanogel technology toward developing biointeractive nanosilver nanogel. It seems that the ecofriendly approach to design nanosystem prevails in future.

## 5 Conclusion

Bioengineering of functional nanogels is the fascinating domain of biomedical engineering with enormous potential to create novel architecture toward dedicated applications in human healthcare systems. Nanosilver has been the material of today for its antimicrobial nature. However it lacks the ability to bind to material surface by chemical and physical bonds. Encapsulating the nanosilver within a nanogel seems to a beautiful idea that may lead to nanoparticles where nanosilver is surrounded by a biocompatible hydrogel matrix. Such a nanoparticle could attach to other surfaces depending on the chemical nature of the hydrogel. State of the art is therefore to design and develop a functional nanosilver nanogel that would be biocompatible and biointeractive in nature. Various routes are available for the encapsulation of silver into nanogels and their composites have been widely used. The nanosilver particles may be encapsulated during the polymerization or in a postpolymerization step to give specific architecture to the bioactive nanogel. It seems that the radiation process is very effective in developing nanogels as this allows the polymerization and the silver reduction simultaneously. Moreover, the nanogel may be chemically designed with very precise functional groups and the base matrix of synthetic in origin. The most innovative feature in today's world is that we have a nanosilver particle that is functional due to the polymer nanolayer around it, which makes it to bind to any surface for infection control. May be the hybrid nanostructure would open up a lot more interesting domain. Therefore, the question is that where are we and where do we have to go? It seems that the functional nanosilver may be the prime requirement and we need to look at the possibilities of creating newer structures with additional features to achieve multifeatured materials. The collaborative efforts of material scientists, bioscientists, and biomedical engineers would continue to develop functional nanomaterials with much wider spectrum of applications. Such biointeractive nanogels would certainly open up a revolution in the world of antimicrobial medical devices leading to enormous control over healthcare expenditures.

## Conflict of Interest

The authors declare no conflict of interest.
